# Supplementary material for: On the forecastability of food insecurity
Source: Sci Rep. 2023 Mar 16;13:2793. doi: 10.1038/s41598-023-29700-y (PMC10038988; doi:10.1038/s41598-023-29700-y)
Supplement: Supplementary file 1 — Supplementary Information. [file 41598_2023_29700_MOESM1_ESM.pdf]

Supplementary Information for *On the forecastability of food insecurity*

Pietro Foini[a], Michele Tizzoni[a,b], Giulia Martini[c], Daniela Paolotti[a], and Elisa Omodei[c,d,*]

[a] *ISI Foundation, Via Chisola 5, 10126, Torino, Italy*
[b] *Department of Sociology and Social Research, University of Trento, Via Verdi, 26 I-38122 Trento, Italy*
[c] *World Food Programme, Research, Assessment and Monitoring Division (RAM), Via Cesare Giulio Viola 68, 00148 Rome, Italy*
[d] *Department of Network and Data Science, Central European University, Quellenstraße 51, A-1100 Vienna, Austria*
[*] *To whom correspondence should be addressed. E-mail:omodeie@ceu.edu*

1 Forecasting food insecurity with secondary information

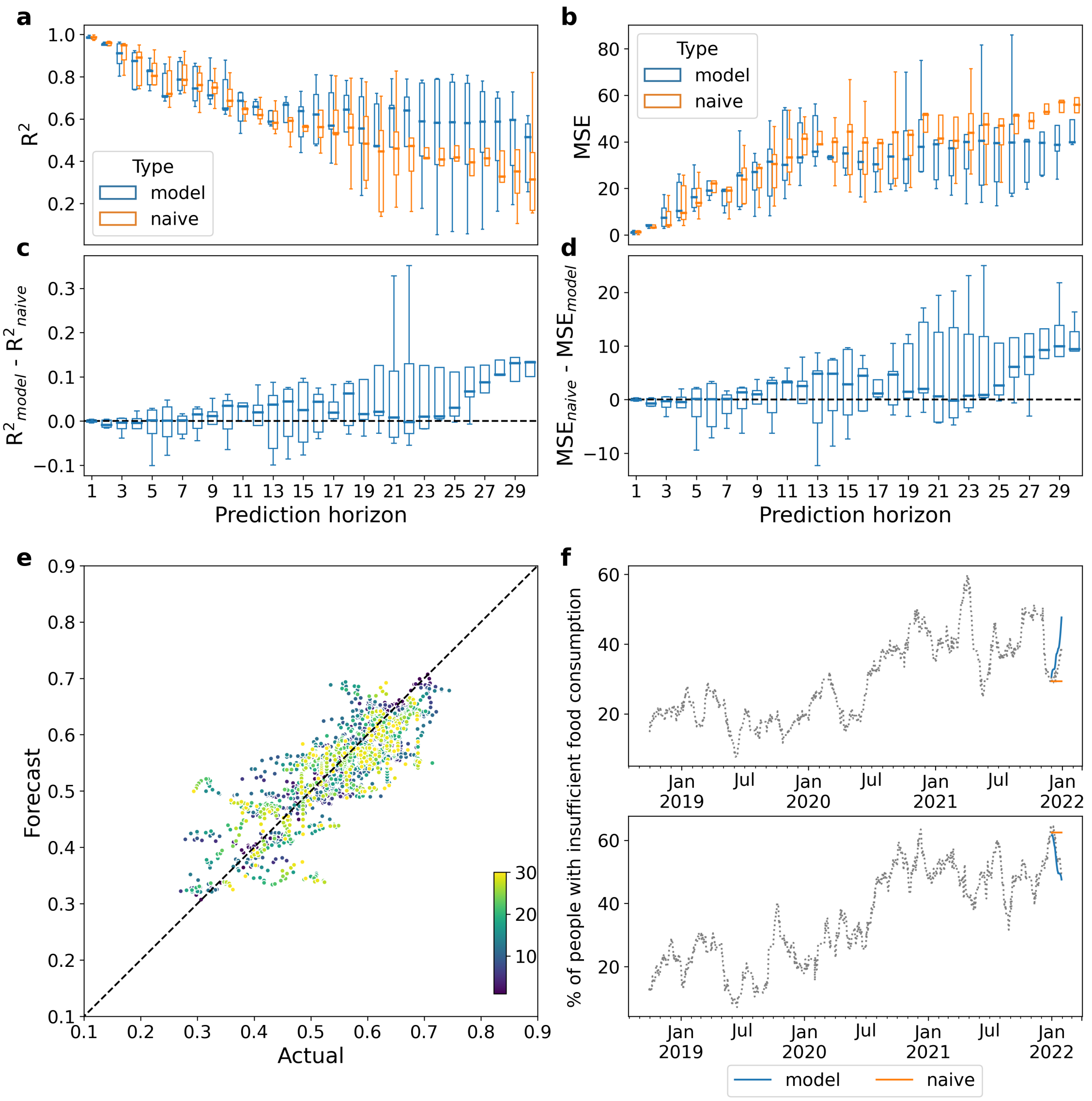

Supplementary Figure 1: **Forecasting the prevalence of people with insufficient food consumption in Syria.** The forecasting is performed over 5 different monthly splits of all governorates time series, from October 2021 to February 2022. (a) Box plots of the coefficient of determinations (R^2) across the 5 splits for both the proposed and the naive models (in blue and orange, respectively), for each forecasting horizon. (b) Box plots of the mean squared error (MSE) across the 5 splits for both the proposed and the naive models for each forecasting horizon. (c) Box plots of the difference between the R^2 of the proposed and of the naive model for each split. (d) Box plots of the difference between the MSE of the naive and of the proposed model for each split. (e) Predicted vs actual value for each data point in the 5 splits. Colors represent the corresponding forecasting horizon and vary from dark blue (1 day) to yellow (30 days) (f) Example of forecasting results for December 2021 in Damascus (top) and January 2022 in Tartous (bottom).

Supplementary Figure 2: **Forecasting the prevalence of people with insufficient food consumption in Burkina Faso.** The forecasting is performed over 5 different monthly splits of all sub-national time-series, from October 2021 to February 2022. (a) Box plots of the coefficient of determinations (R^2) across the 5 splits for both the proposed and the naive models (in blue and orange, respectively), for each forecasting horizon. (b) Box plots of the mean squared error (MSE) across the 5 splits for both the proposed and the naive models for each forecasting horizon. (c) Box plots of the difference between the R^2 of the proposed and of the naive model for each split. (d) Box plots of the difference between the MSE of the naive and of the proposed model for each split. (e) Predicted vs actual value for each data point in the 5 splits. Colors represent the corresponding forecasting horizon and vary from dark blue (1 day) to yellow (30 days) (f) Example of forecasting results for December 2021 in Boucle-Du-Mouhoun (top) and January 2022 in Centre-Ouest (bottom).

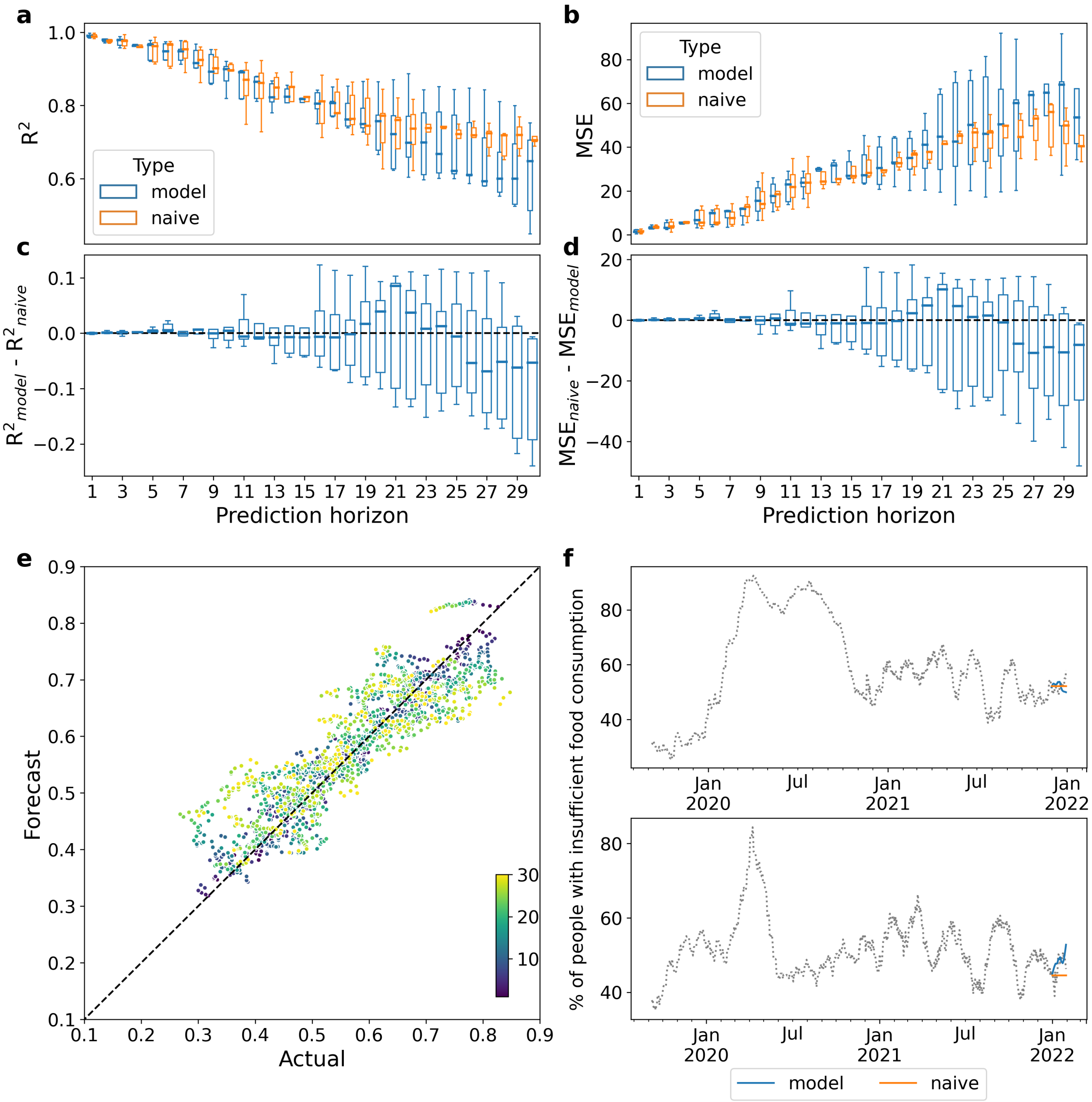

Supplementary Figure 3: **Forecasting the prevalence of people with insufficient food consumption in Mali.** The forecasting is performed over 3 different monthly splits of all sub-national time-series, from October 2021 to February 2022. (a) Box plots of the coefficient of determinations (R^2) across the 5 splits for both the proposed and the naive models (in blue and orange, respectively), for each forecasting horizon. (b) Box plots of the mean squared error (MSE) across the 5 splits for both the proposed and the naive models for each forecasting horizon. (c) Box plots of the difference between the R^2 of the proposed and of the naive model for each split. (d) Box plots of the difference between the MSE of the naive and of the proposed model for each split. (e) Predicted vs actual value for each data point in the 5 splits. Colors represent the corresponding forecasting horizon and vary from dark blue (1 day) to yellow (30 days) (f) Example of forecasting results for February 2022 in Koulikoro (top) and December 2021 in Sikasso (bottom).

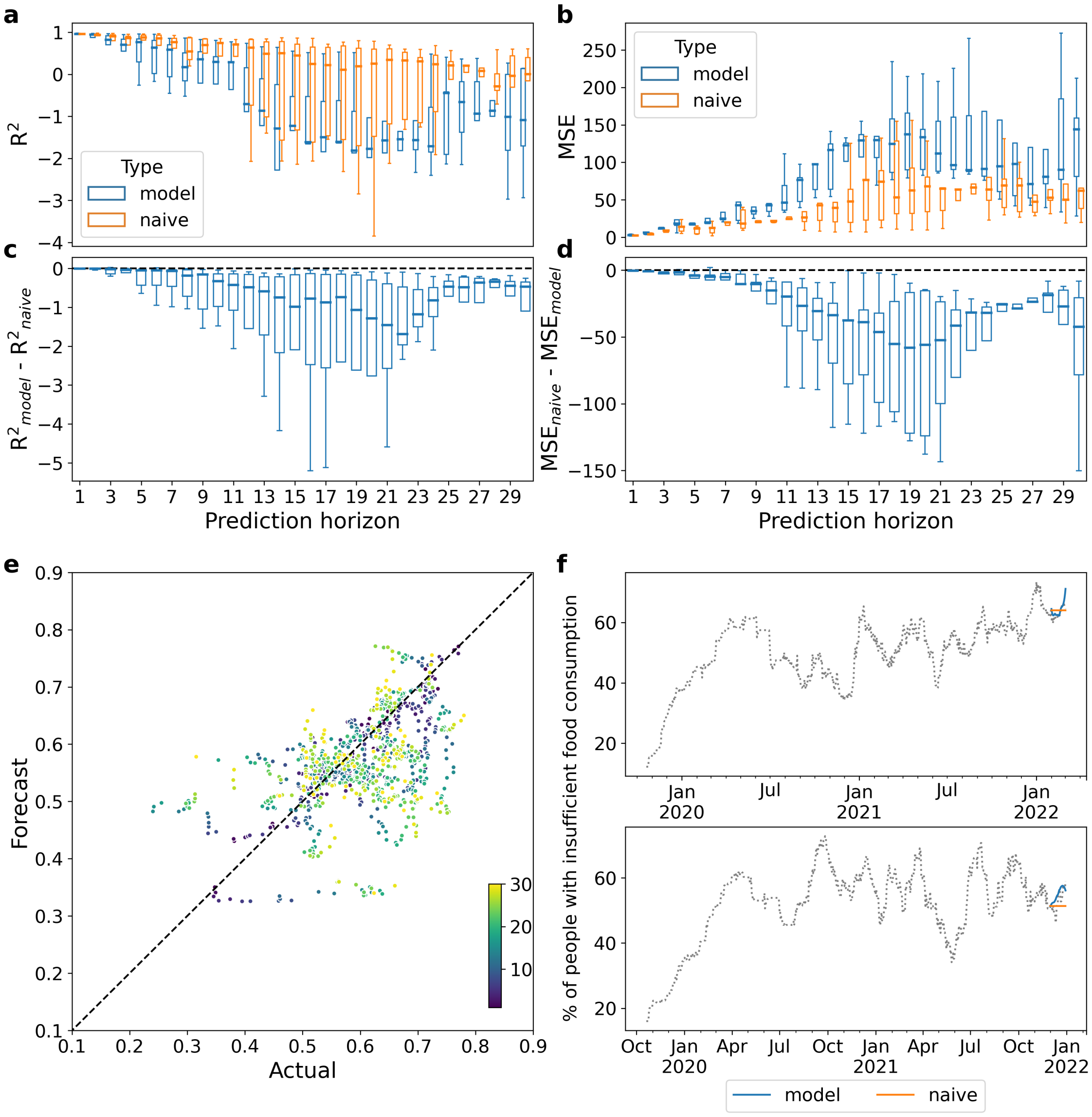

Supplementary Figure 4: **Forecasting the prevalence of people with insufficient food consumption in Cameroon.** The forecasting is performed over 5 different monthly splits of all sub-national time-series, from October 2021 to February 2022. (a) Box plots of the coefficient of determinations (R^2) across the 5 splits for both the proposed and the naive models (in blue and orange, respectively), for each forecasting horizon. (b) Box plots of the mean squared error (MSE) across the 5 splits for both the proposed and the naive models for each forecasting horizon. (c) Box plots of the difference between the R^2 of the proposed and of the naive model for each split. (d) Box plots of the difference between the MSE of the naive and of the proposed model for each split. (e) Predicted vs actual value for each data point in the 5 splits. Colors represent the corresponding forecasting horizon and vary from dark blue (1 day) to yellow (30 days) (f) Example of forecasting results for February 2022 in Central (top) and December 2021 in North-West (bottom).

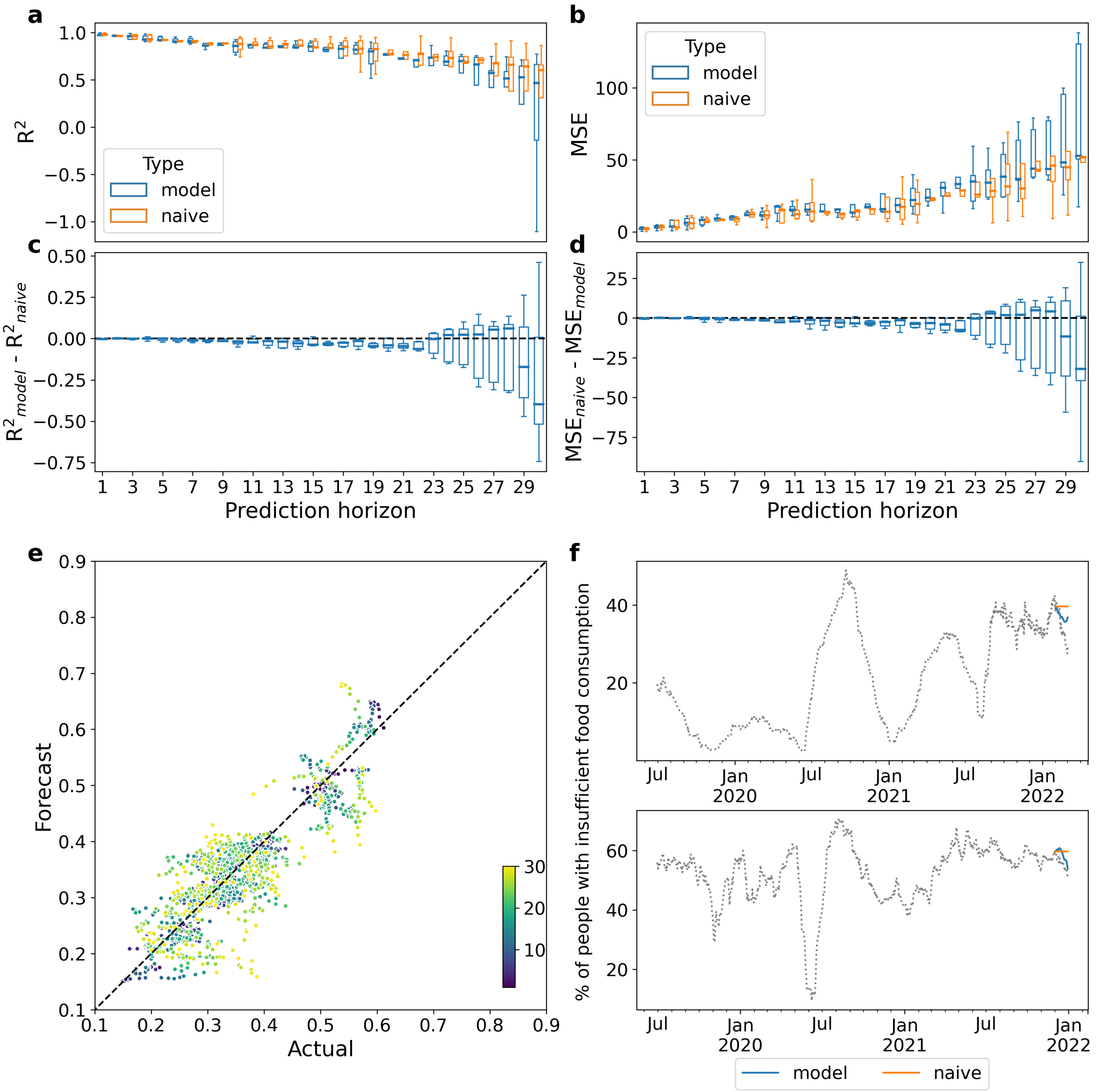

Supplementary Figure 5: **Forecasting the prevalence of people with insufficient food consumption in Nigeria.** The forecasting is performed over 5 different monthly splits of all sub-national time-series, from October 2021 to February 2022. (a) Box plots of the coefficient of determinations (R^2) across the 5 splits for both the proposed and the naive models (in blue and orange, respectively), for each forecasting horizon. (b) Box plots of the mean squared error (MSE) across the 5 splits for both the proposed and the naive models for each forecasting horizon. (c) Box plots of the difference between the R^2 of the proposed and of the naive model for each split. (d) Box plots of the difference between the MSE of the naive and of the proposed model for each split. (e) Predicted vs actual value for each data point in the 5 splits. Colors represent the corresponding forecasting horizon and vary from dark blue (1 day) to yellow (30 days) (f) Example of forecasting results for January 2022 in Yobe (top) and January 2022 in Borno (bottom).

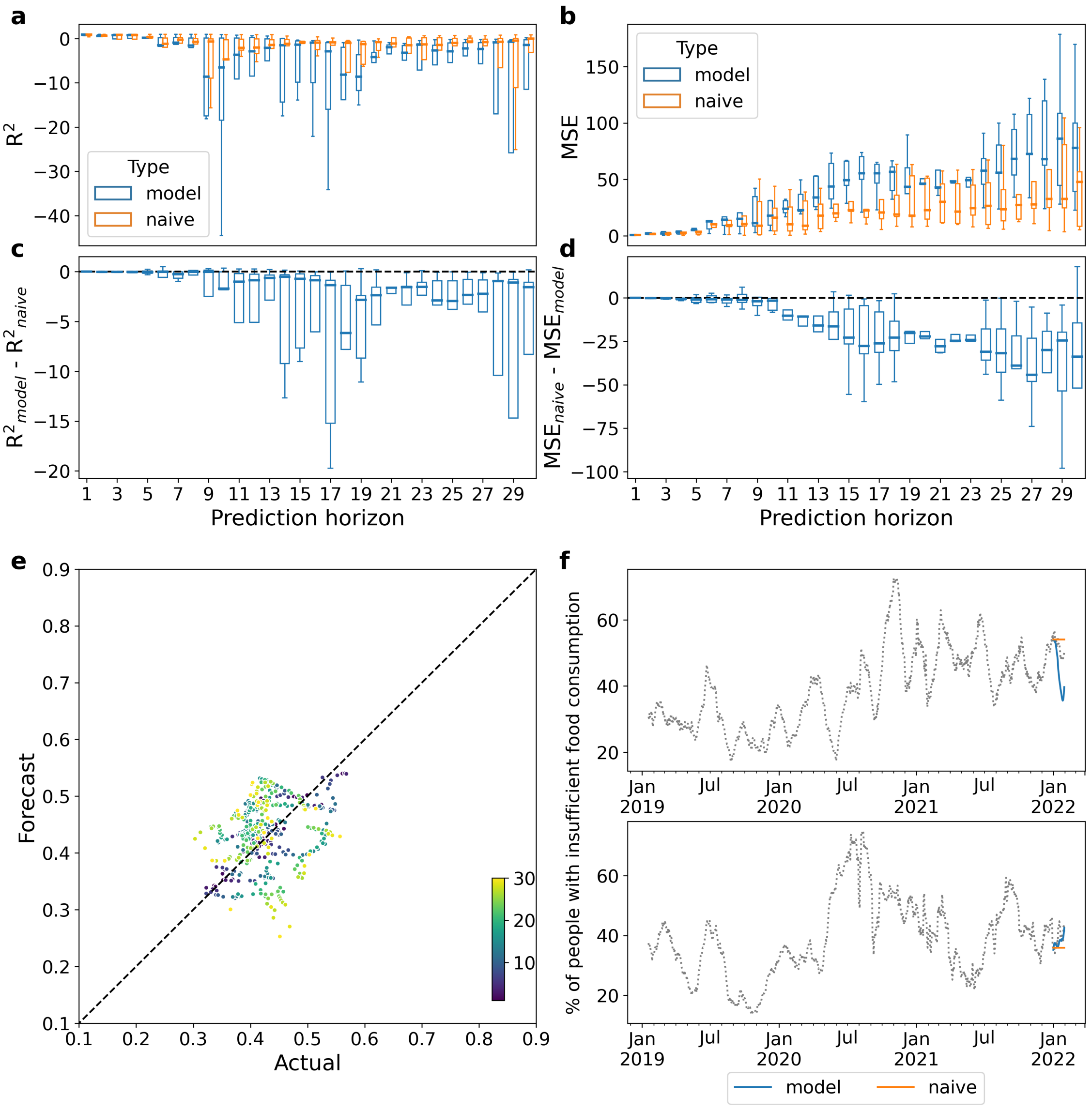

2 Model performance as a function of data availability

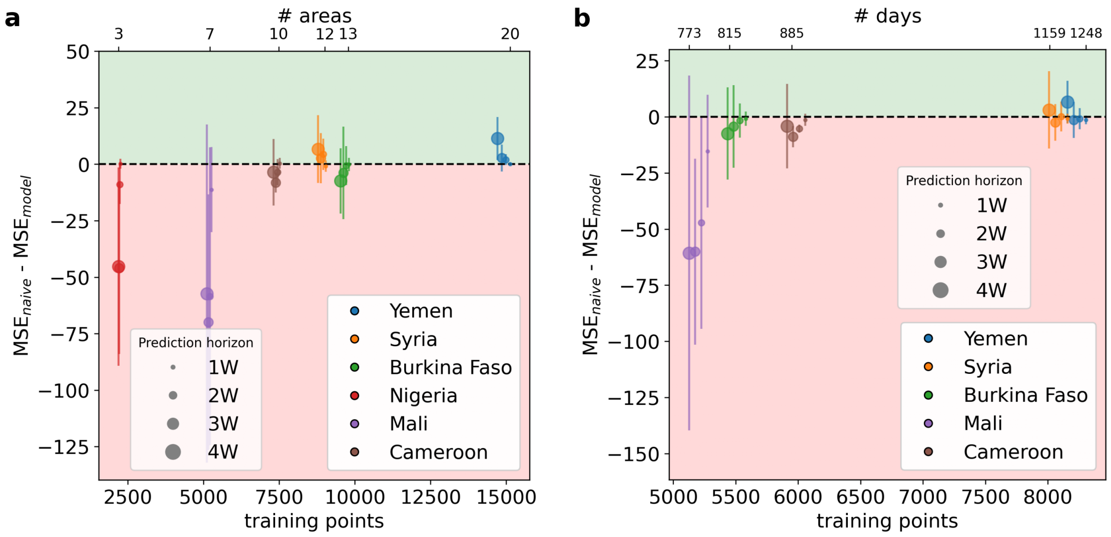

Supplementary Figure 6: **Model performance as a function of different dimensions.** Panel (a) shows the averaged differences between the MSE of the naive approach and the MSE of the forecasting model across the different splits, as a function of the number of training points obtained by fixing the same time series length for all countries. Error bars correspond to the relative standard deviation. The green area indicates where our model outperforms the naive one, the red area indicates the opposite. Panel (b) shows the case in which we instead considered the full time series but fixed the number of considered areas to be the same for all countries.

3 Data Sources

In this Section, we describe in details all the data sources used in this study.

3.1 Prevalence of people with insufficient food consumption

The prevalence of individuals with insufficient food consumption is estimated from one of the most commonly used food security indicators, the Food Consumption Score (FCS), which represents households' dietary diversity and nutrient intake [1].

The FCS is based on household surveys containing questions about quantity and frequency of consumption of food items from different food groups during a 7-day reference period. For each household, food groups consumption frequencies are then summed up in a weighted fashion, with more nutritious foods having higher weights:

$$FCS_{household} = \sum_{i=1}^{nb\ food\ groups} w_i x_i \tag{1}$$

where w_i is the weight of food group i (see Table 1) and x_i represents the number of days the household has consumed items from food group i.

Each household is considered to have poor, borderline or acceptable food consumption based on the value of its FCS: households with FCS $\leq$ 21 are labelled as having a poor food consumption profile, households with 21 $<$ FCS $\leq$ 35 a borderline profile, and households with FCS $>$ 35 have an adequate food consumption profile. These thresholds have been defined based on assumptions on dietary patterns, but might be adapted on a per case basis to better reflect local contexts [1].

In order to obtain the prevalence of people with insufficient food consumption for a given geographical area and time window, a representative number of surveys is performed and the prevalence of households with poor or borderline food consumption is estimated. The data used in this study have been collected by WFP by means of daily remote phone surveys. The prevalence of people with insufficient food consumption for a given date is obtained by considering households interviewed during the previous d days, where d assumes different values in different geographical areas, as reported in Table 2.

The start and end dates of the time series analyzed in this study are reported, for each country, in Table 3. Missing daily values are inferred through linear interpolation (i.e. interpolating between the value for the previous day for the area under consideration and the corresponding value for the following day) but sub-national time series presenting more than 7 consecutive missing values (i.e. one week) are discarded. Table 4 reports the final list of first-level administrative units considered for each country, the overall population that they cover, and what share of the country population it corresponds to.

3.2 Prevalence of people using crisis or above crisis food-based coping

The prevalence of people using crisis or above crisis food-based coping is derived from another popular food security indicator, the reduced Coping Strategy Index (rCSI) [2].

Supplementary Table 1: Food groups and corresponding weights used to compute the Food Consumption Score [1].

**Food group**	**Weight**
Main staples	2
Pulses	3
Vegetables	1
Fruit	1
Meat and fish	4
Milk	4
Sugar	0.5
Oil	0.5
Condiments	0

Supplementary Table 2: Time windows (i.e. number of days) used to obtain the prevalence of people with insufficient food consumption analyzed in this study for Burkina Faso (BFA), Syria (SYR), Yemen (YEM), Cameroon (CMR), Mali (MLI), and Nigeria (NGA). Some countries have multiple numbers because different time windows are used for different sub-national areas.

	d
**YEM**	31
**SYR**	31
**BFA**	56, 30
**CMR**	30, 90
**MLI**	28, 84, 30
**NGA**	31, 30

Supplementary Table 3: Start and end dates of the time series of the prevalence of people with insufficient food consumption analyzed in this study.

	**Start**	**End**
**YEM**	2018-07-02	2022-03-02
**SYR**	2018-08-31	2022-03-02
**BFA**	2019-09-08	2022-03-02
**CMR**	2019-06-30	2022-03-02
**MLI**	2019-10-20	2022-03-02
**NGA**	2019-01-18	2022-03-02

The rCSI is based on household surveys containg questions about how often households had to adopt one of more strategies to cope with not having enough food or enough money to buy food, during a 7-day reference period. For each household, coping strategies are then summed up in a weighted fashion to determine the corresponding rCSI. The five inquired strategies and their relative weights are reported in Table 5.

In order to obtain the prevalence of people using crisis or above crisis food-based coping for a given geographical area and time window, a representative number of surveys is performed and the prevalence of households with rCSI ≥ 19 is considered, as defined by the Integrated Food Security Phase Classification framework [3].

Supplementary Table 4: Administrative regions covered by the time series analyzed in this study.

Country	Administrative Regions	Population covered (Millions)	Share of total population
**YEM**	Abyan, Al Hudaydah, Al Jawf, Al Maharah, Al Mahwit, Amanat Al Asimah, Amran, Dhamar, Hajjah, Ibb, Lahj, Marib, Raymah, Sa'ada, Sana'a, Shabwah, Taizz, Aden, Al Bayda, Al Dhale'e	28.5	95%
**SYR**	Tartous, Al-Hasakeh, Aleppo, As-Sweida, Damascus, Dar'a, Hama, Homs, Lattakia, Rural Damascus, Ar-Raqqa, Deir-ez-Zor	18.0	88%
**BFA**	Boucle-Du-Mouhoun, Cascades, Centre, Centre-Est, Centre-Nord, Centre-Ouest, Centre-Sud, Est, Hauts-Bassins, Nord, Plateau-Central, Sahel, Sud-Ouest	19.7	100%
**CMR**	Adamawa, Central, East, Far-North, Littoral, North, North-West, South-West, West	24.4	97%
**MLI**	Bamako, Kayes, Koulikoro, Mopti, Segou, Sikasso, Tombouctou-Taoudeni	18.3	96%
**NGA**	Adamawa, Borno, Yobe	15.2	7%

The survey data used in this study for the prevalence of people using crisis or above crisis food-based coping are the same as for the prevalence of people with insufficient food consumption, hence all the information reported in the previous section on the time series spatial and temporal resolution apply for this indicator too. Furthermore, Yemen's March 2019 values were all replaced by means of linear interpolation (i.e. interpolating between the value for the previous day for the area under consideration and the corresponding value for the following day) because of some anomalies in the original data. Fig. 7 shows all the food-based coping time series analyzed in this study.

Supplementary Table 5: Coping strategies asked to build the rCSI indicator and their corresponding weights [2].

Coping strategy	Severity weight
Rely on less preferred or less expensive food	1
Borrow food or rely on help from friends or relatives	2
Limit portion size at mealtimes	1
Restrict consumption by adults in order for small children to eat	3
Reduce number of meals eaten in a day	1

Supplementary Figure 7: **Time trends of crisis or above crisis food-based coping.** Each panel displays daily time series of the percentage of people using crisis or above crisis food-based coping in the first-level administrative units of Burkina Faso, Cameroon, Mali, Nigeria, Syria and Yemen analyzed in this study.

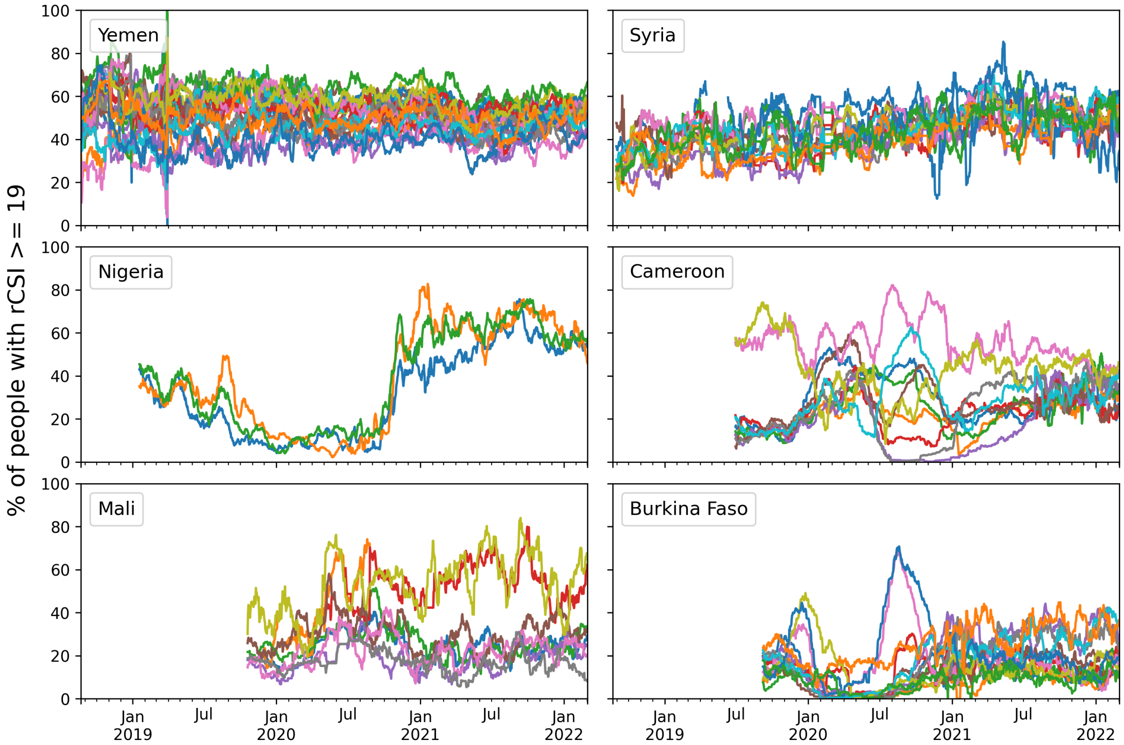

3.3 Conflict-related fatalities

The Armed Conflict Location & Event Data Project (ACLED) is a publicly available repository providing real-time and historical data on political violence and protest events in nearly 100 countries [4]. Data about conflicts are made available each week after curation from individual researchers collecting information from available reports. Information is also provided with respect to the date (when the event happened), type of violence (what happened), actors (who is involved), and location (where the event happened). Moreover, for each event, the estimated number of fatalities is reported (ACLED does not independently verify reported fatalities, and includes this information as an estimate only, reflecting the content of media reports).

From this data, we build daily time series for each analyzed region and date by summing all the reported fatalities in a given region during the previous d days. If on a given day no event is recorded by ACLED for a given region then we assume zero fatalities in that region for that day. The time series thus obtained are shown in Fig. 8.

Supplementary Figure 8: **Time trends of conflict-related fatalities.** Each panel displays daily time series of conflict-related fatalities in the first-level administrative units of Burkina Faso, Cameroon, Mali, Nigeria, Syria and Yemen analyzed in this study.

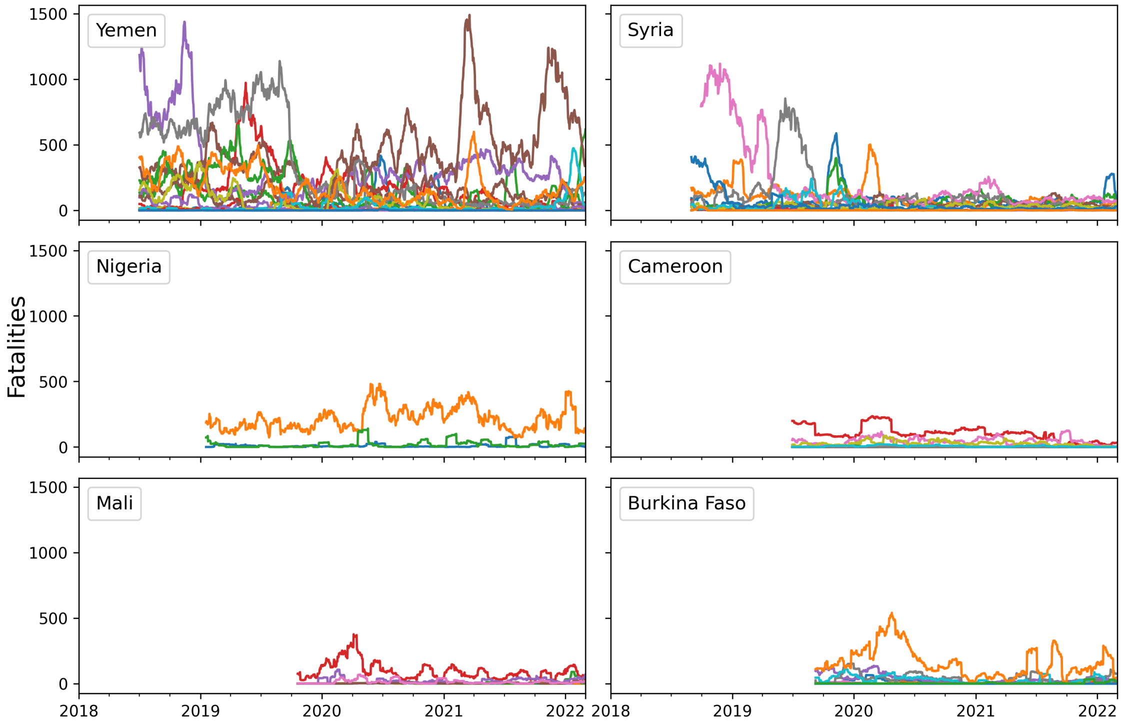

3.4 Market prices

WFP monitors commodity prices in local markets on a monthly basis, and makes them available through its Economic Explorer (https://dataviz.vam.wfp.org/economic_explorer/prices).

Commodities are categorized in eight groups ('cereals and tubers', 'meat, fish and eggs', 'milk and dairy', 'miscellaneous food', 'non-food', 'oil and fats', 'pulses and nuts' and 'vegetables and fruits'). In this study, we consider commodities in the 'cereals and tubers' category (which include rice, bulgur, millet, sorghum, etc.), since they are widely consumed food across all the different geographical contexts analyzed.

As some administrative regions can include multiple markets, we first average the price of a given commodity across all markets in the region under consideration. We then normalize all prices between 0 and 1 to discard differences in prices across different commodities (since the relevant information is whether prices are increasing or decreasing, rather than their absolute value) and finally average all normalized prices across the different commodities in each region. This allows us to have a final unique time series describing cereal and tubers price variations within each region. Missing monthly values (up to a maximum of 3 months) are inferred through linear interpolation (i.e. interpolating between the value for the previous month for the area under consideration and the corresponding value for the following month). For Cameroon, data gaps were too extensive and the variable was therefore discarded. The time series thus obtained are shown in Fig. 9.

Supplementary Figure 9: **Time trends of averaged and normalized market prices of cereals and tubers.** Each panel displays daily time series of averaged and normalized market prices of cereals and tubers in the first-level administrative units of Burkina Faso, Mali, Nigeria, Syria and Yemen analyzed in this study. Cameroon is not included due to the presence of large data gaps.

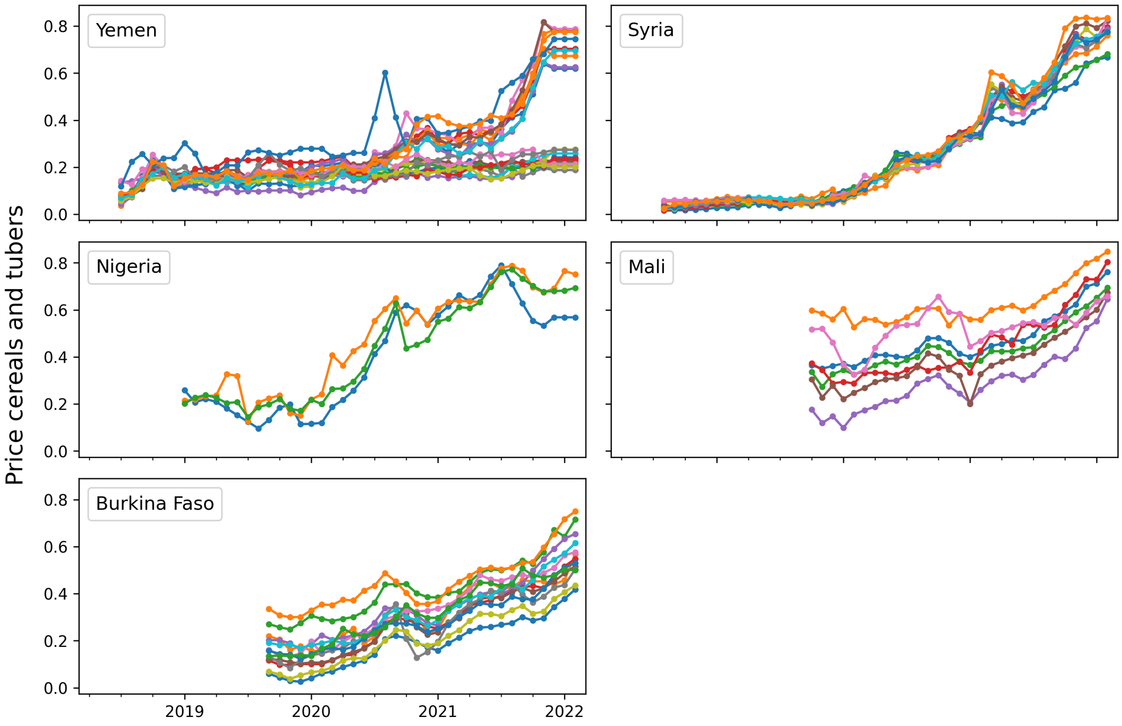

3.5 Weather-related variables

WFP provides a publicly available platform called Seasonal Explorer (https://dataviz.vam.wfp.org/seasonal_explorer/rainfall_vegetation/visualizations) allowing users to assess sub-national near-global information on the current and past rainfall seasons such as the timing and intensity of drier or wetter than average conditions and their impact on vegetation status. The primary data sources are CHIRPS gridded rainfall dataset produced by the Climate Hazards Group at the University of California, Santa Barbara [5] and the MODIS NDVI CMG data made available by NOAA-NASA [6].

Data are reported for each administrative region for each 10-day window of the month ('dekad') for the following indicators: rainfall amount (in mm), 1-month rainfall anomaly, 3-month rainfall anomaly, normalized difference vegetation index (NDVI), and NDVI anomaly. The latter is meant in comparison with the long term average: for example, a value of 73% of the 3-month rainfall anomaly in the second half of August means that the amount of rainfall for the three month period ending on August 20th has been 73% of the average amount of rainfall measured in the same area during the same period of the year. The corresponding time series are shown in Figs. 10, 11, 12, 13, 14.

Supplementary Figure 10: **Time trends of rainfall.** Each panel displays dekad time series of rainfall in the first-level administrative units of Burkina Faso, Mali, Nigeria, Syria and Yemen analyzed in this study.

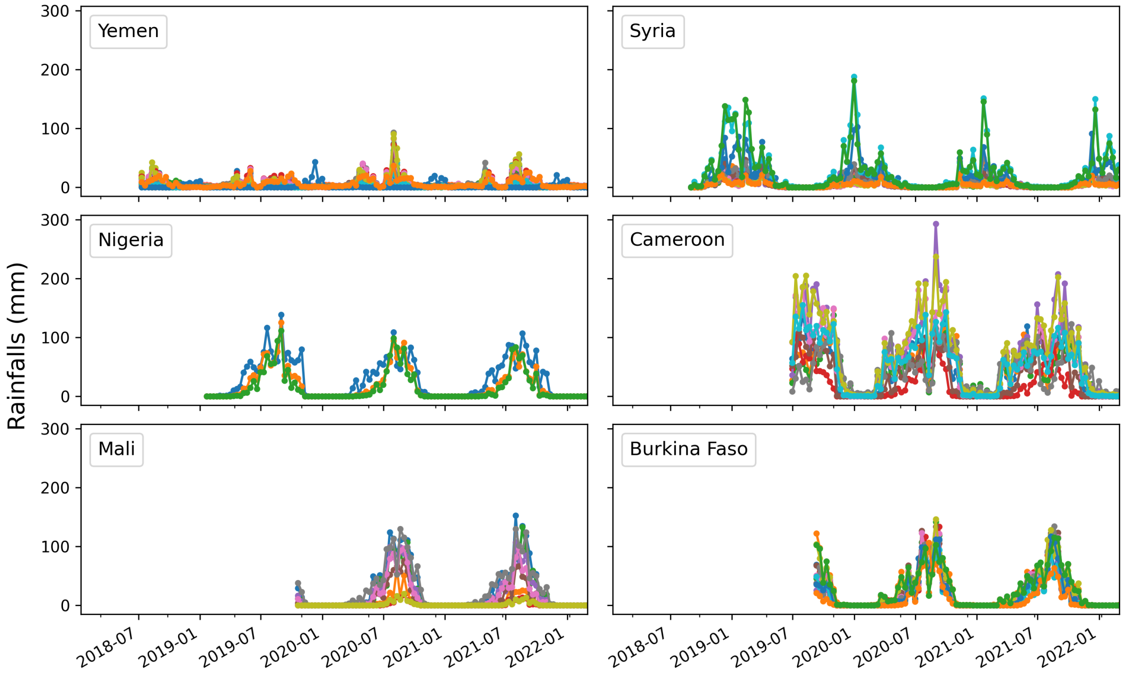

Supplementary Figure 11: **Time trends of 1-month rainfall anomaly.** Each panel displays dekad time series of 1-month rainfall anomaly in the first-level administrative units of Burkina Faso, Mali, Nigeria, Syria and Yemen analyzed in this study.

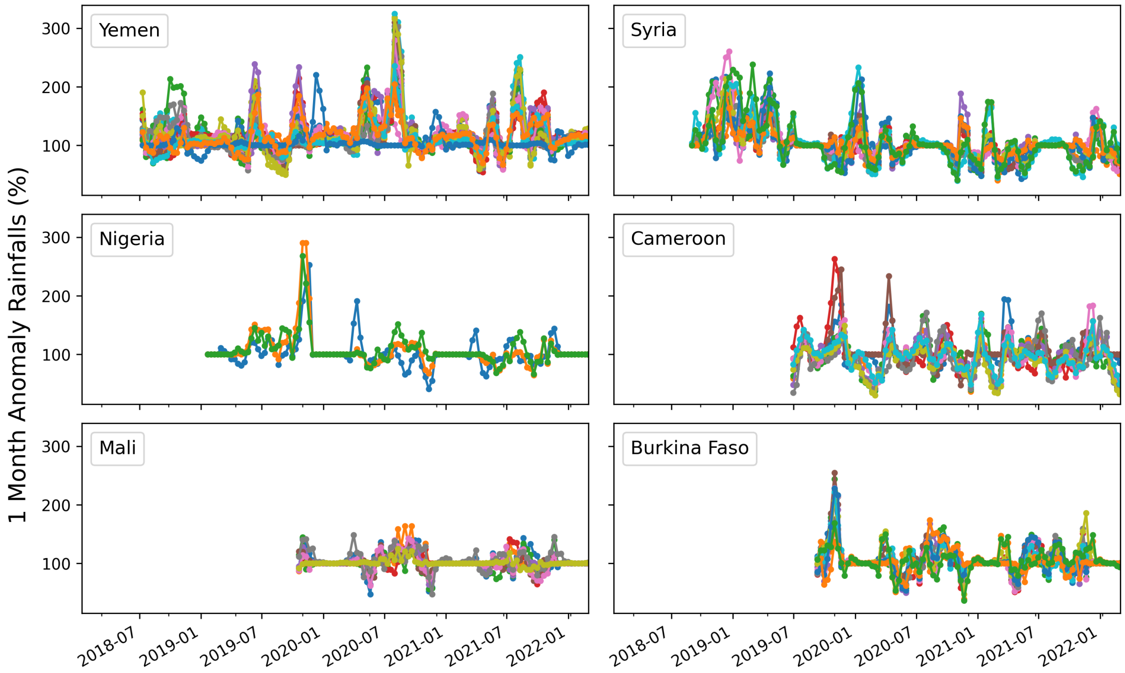

Supplementary Figure 12: **Time trends of 3-month rainfall anomaly.** Each panel displays dekad time series of 3-month rainfall anomaly in the first-level administrative units of Burkina Faso, Mali, Nigeria, Syria and Yemen analyzed in this study.

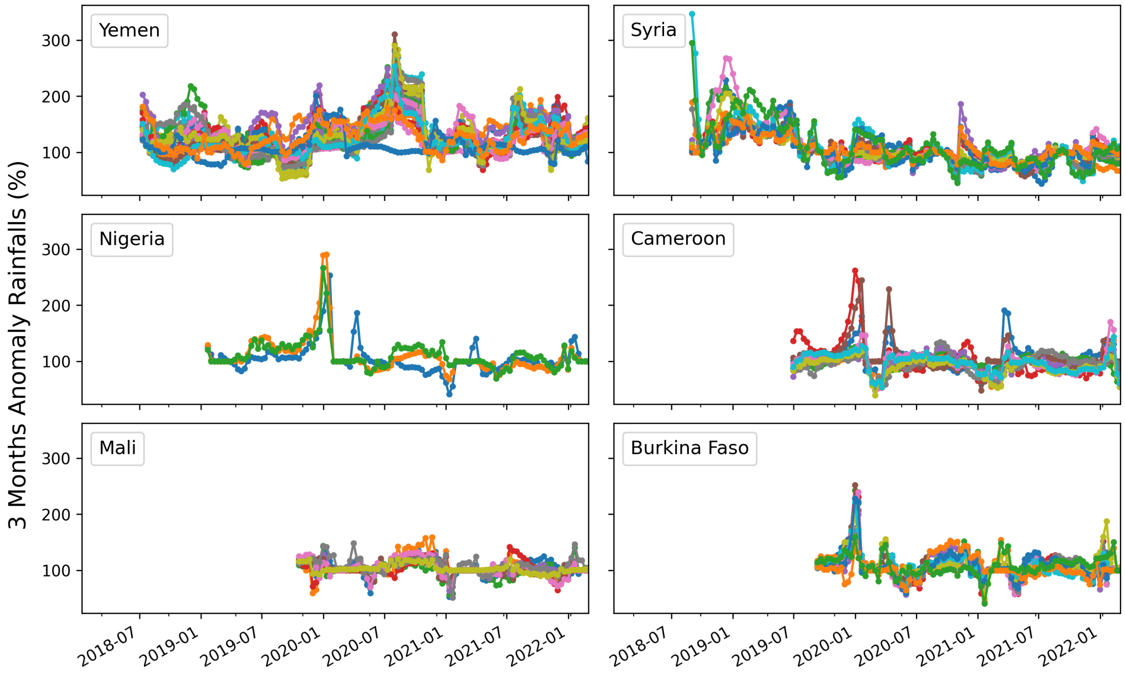

Supplementary Figure 13: **Time trends of NDVI.** Each panel displays dekad time series of NDVI in the first-level administrative units of Burkina Faso, Mali, Nigeria, Syria and Yemen analyzed in this study

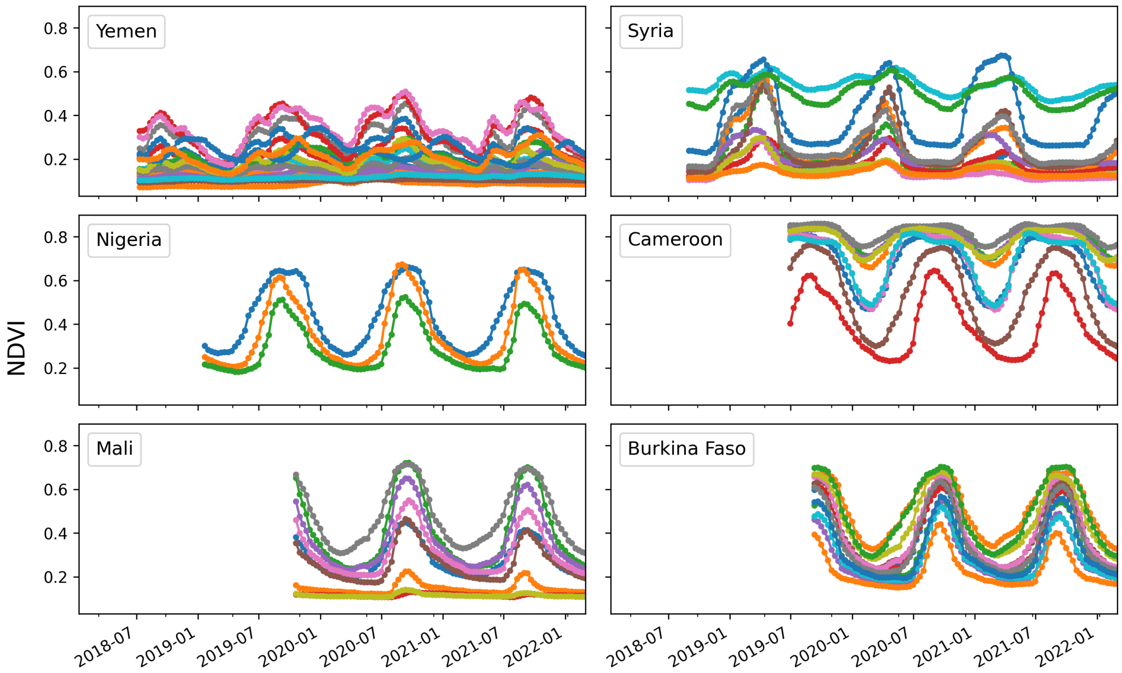

Supplementary Figure 14: **Time trends of NDVI anomaly.** Each panel displays dekad time series of NDVI anomaly in the first-level administrative units of Burkina Faso, Mali, Nigeria, Syria and Yemen analyzed in this study.

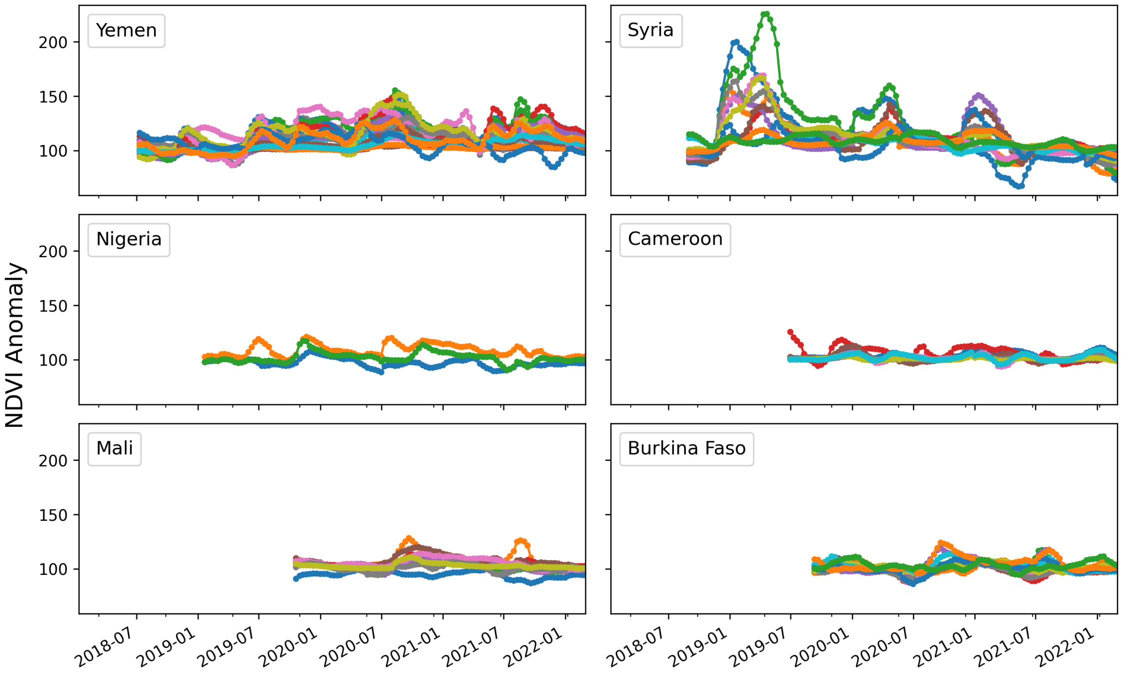

4 Permutation Entropy

A preliminary analysis of how predictable insufficient food consumption time series are can support the decision regarding the most suitable forecasting approach. To this aim, we use the *Permutation Entropy* (PE) as a model-free measure of time series predictability [7]. This approach is based on measuring the Shannon entropy of a time series by means of estimating the probabilities of observing trend patterns within the time series itself. The PE approach thus categorizes the continuous time series X in a small set of symbols or alphabet according to their trends.

4.1 Symbolization procedure

The first stage of the PE converts a continuous time series X into a sequence of discrete symbols $\hat{X}$ by adopting a permutation technique. Let $x(i)$ $i = 1, ..., N$ denote sequences of observations from systems X. For a given, but otherwise arbitrary i, m amplitude values $X_i = \{x(i), x(i+\tau), \ldots, x(i+(m-1)\tau)\}$ are arranged in an ascending order where τ denotes the time delay, and m is the embedding dimension. In the unlikely case of equal amplitude, the observations are ordered according to their original index. Therefore, each X_i is mapped onto one of the $m!$ possible permutations.

Let's suppose to have a test time series $X = \{120, 74, 203, 167, 92, 148, 174, 47\}$ and to transform it into a symbolic sequence. For simplicity, let's take the embedding dimension $m = 3$ and $\tau = 1$. This quantity determines the amount of symbols that can possibly exist, more precisely $m!$. The first step to transform X into a symbolized time series is to slide a window of length m over the time series. So, we start taking the first three elements of X and we sort them in increasing order, which leaves us with $\{74, 120, 203\}$. Now, we keep tract of these values' indices, such that the sequence looks like $\{2, 1, 3\}$. This first subchain maps to symbol D. From this scheme, we just need to advance the window one value at a time: the next sub-chain to consider is $\{74, 203, 167\}$. Its sorted version is $\{74, 167, 203\}$ which corresponds to $\{1, 3, 2\}$ pattern, and maps to B. And so on, until to achieve the final symbolic sequence $\hat{X} = \{D, B, F, E, A, C\}$.

4.2 Shannon entropy

The PE of the time series X is given by the Shannon entropy on the permutation orders:

$$H = -\sum_{\pi} p_\pi log(p_\pi) \tag{2}$$

where p_π is the probability of encountering the pattern associated with permutation π. An important convenience of symbolic approaches is that they discount the relative magnitude of the time series [8]. This is important in our case because different geographical areas can differ largely in the prevalence of people with insufficient food consumption. The embedding dimension m and the time delay τ are to be set in order to derive a reliable state space. There exist different procedural approaches in order to deal with this setting decision [9, 10]. In order to find the appropriate embedding dimension for clustering a set of time series, we follow the instructions proposed by Scarpino & Petri [7]. The time delay is fixed to $\tau = 1$ in order to

Supplementary Figure 15: Correlation analysis for Yemen.

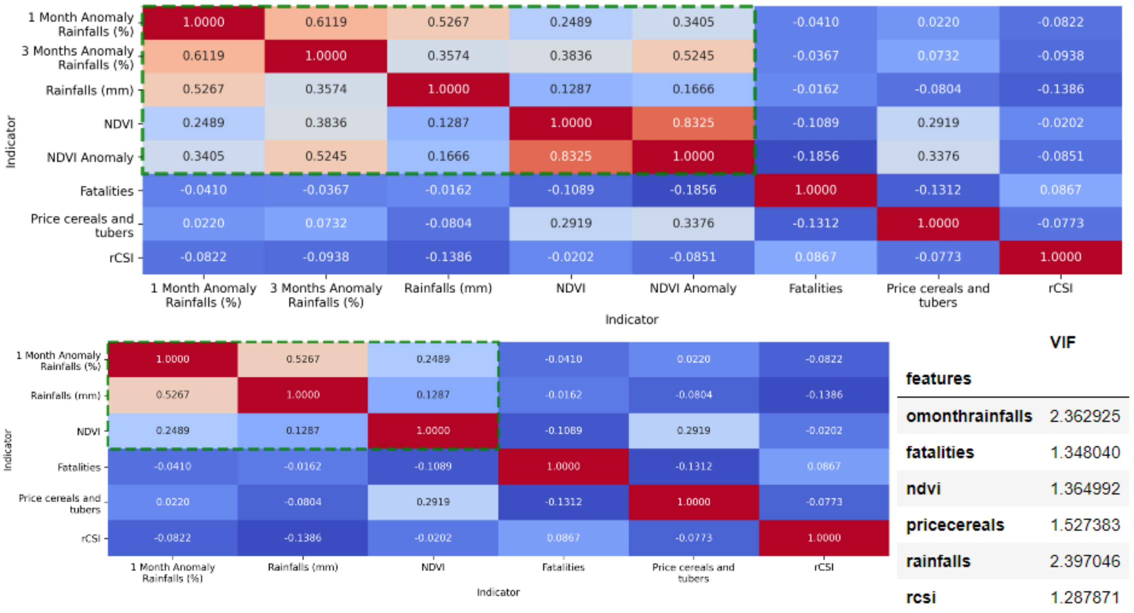

get results from continuous intervals. Finally, the metric used is the predictability defined as $\chi = 1 - H$. The closer to 1 the χ is, the more regular and predictable the time series is. Contrarily, the smaller χ is, the more noisy and random the time series is.

5 Correlation Analysis

In order to determine, for each country, the most representative independent variables among the five weather-related ones (rainfall, 1-month rainfall anomaly, 3-month rainfall anomaly, NDVI and NDVI anomaly), we have performed some correlation analysis. To this goal, we use the Pearson's correlation coefficient. These measures are calculated by first converting the time series that are not at daily resolution to a daily resolution: we use different interpolation strategies depending on the indicator (e.g. backfilling in case of cereals and tubers prices) and we aggregate them on the basis of the d parameter (e.g. we average the interpolated price of cereals and tubers of the previous d days). Finally, we average the values of the correlations obtained between the different time series across the regions of the same country. The results of this correlation analysis are shown in Figs. 15, 16, 17, 18, 19, 20. All cases where the pairwise correlation was above 0.45 were then considered. In each case, one of the two variables was discarded to avoid collinearity, as listed in Table 6. This table also reports the Variance Inflation Factor (VIF) was computed for all remaining variables. All values are all below 3, indicating no significant multicollinearity.

Supplementary Figure 16: Correlation analysis for Syria.

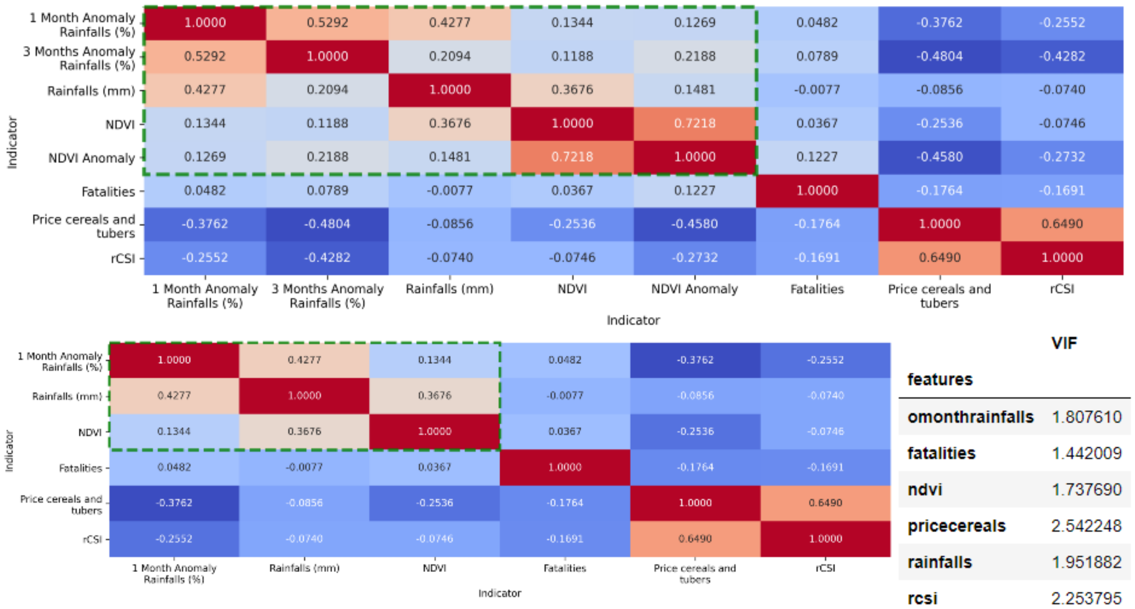

features	VIF
omonthrainfalls	1.807610
fatalities	1.442009
ndvi	1.737690
pricecereals	2.542248
rainfalls	1.951882
rcsi	2.253795

Supplementary Figure 17: Correlation analysis for Nigeria.

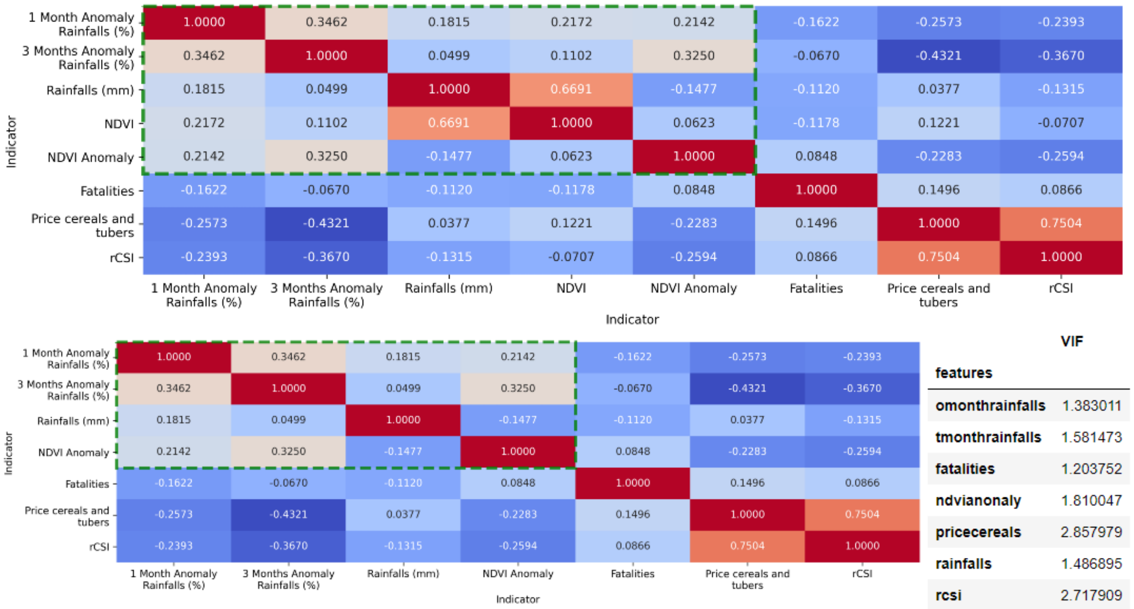

features	VIF
omonthrainfalls	1.383011
tmonthrainfalls	1.581473
fatalities	1.203752
ndvianonaly	1.810047
pricecereals	2.857979
rainfalls	1.486895
rcsi	2.717909

Supplementary Table 6: List of indicators discarded by country as a result of the correlation analysis.

Country	Discarded indicators
YEM	NDVI Anomaly, 3 Months Anomaly Rainfalls
SYR	NDVI Anomaly, 3 Months Anomaly Rainfalls
BFA	NDVI, 3 Months Anomaly Rainfalls
CMR	NDVI, 3 Months Anomaly Rainfalls
MLI	NDVI, 1 Month Anomaly Rainfalls
NGA	NDVI

Supplementary Figure 18: Correlation analysis for Burkina Faso.

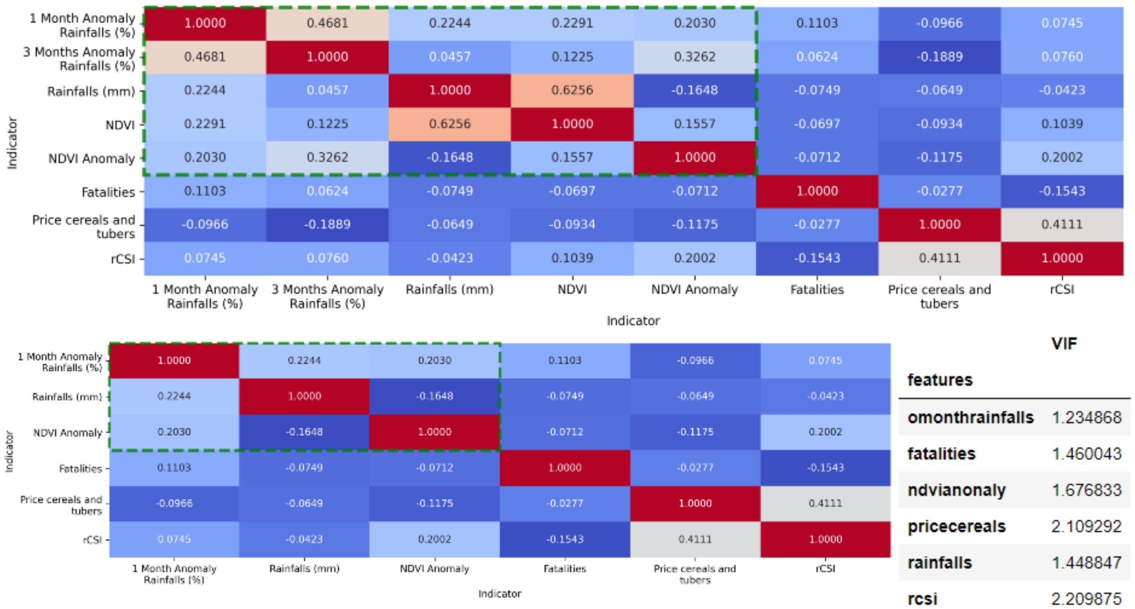

features	VIF
omonthrainfalls	1.234868
fatalities	1.460043
ndvianonaly	1.676833
pricecereals	2.109292
rainfalls	1.448847
rcsi	2.209875

Supplementary Figure 19: Correlation analysis for Mali.

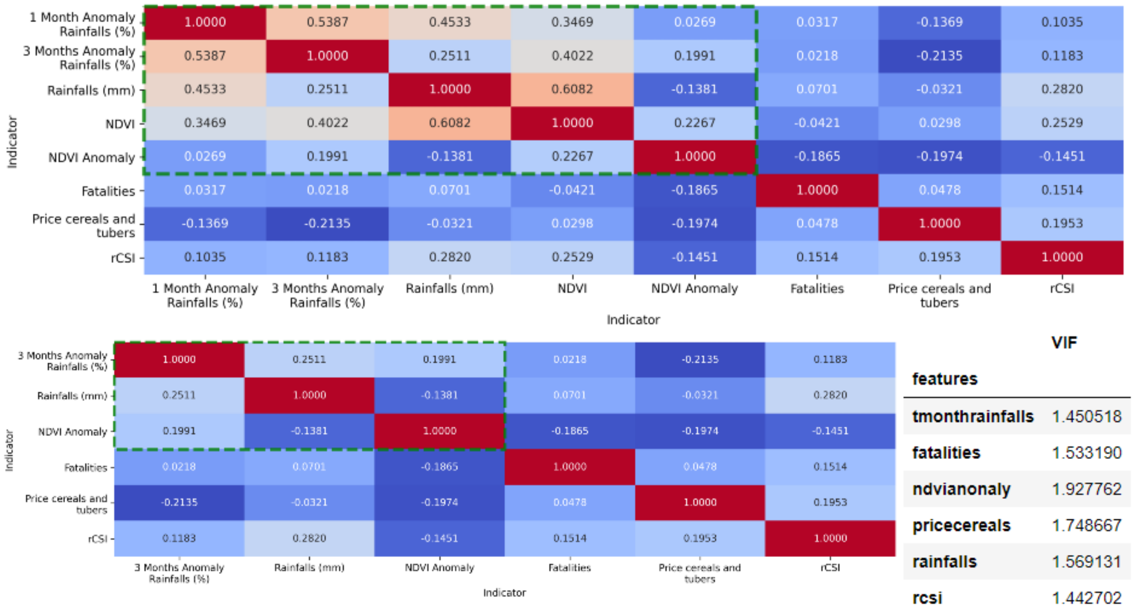

features	VIF
tmonthrainfalls	1.450518
fatalities	1.533190
ndvianonaly	1.927762
pricecereals	1.748667
rainfalls	1.569131
rcsi	1.442702

Supplementary Figure 20: Correlation analysis for Cameroon.

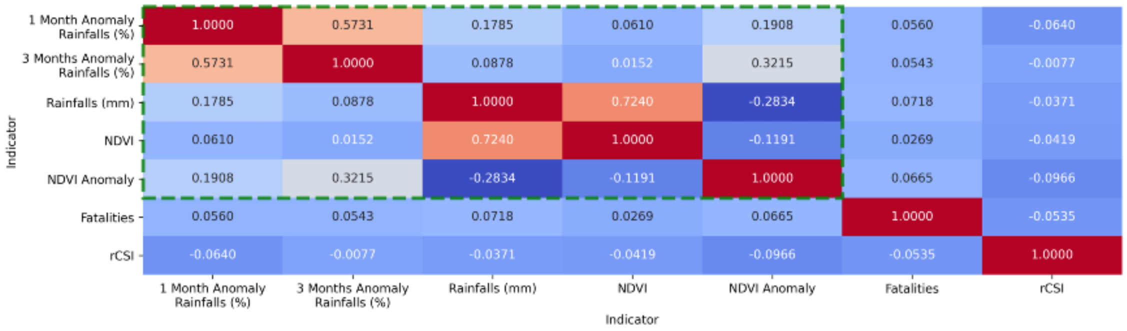

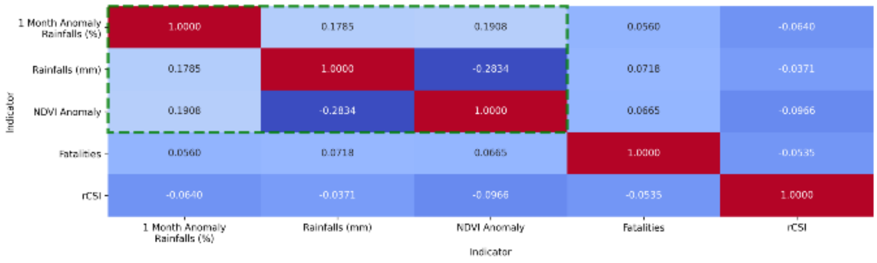

	VIF
**features**	
**omonthrainfalls**	1.304709
**fatalities**	1.190897
**ndvianonaly**	1.466029
**rainfalls**	1.522859
**rcsi**	1.148549

6 Forecasting

The forecasting effort focuses on predicting daily sub-national prevalence of people with insufficient food consumption up to 30 days in advance. There exists a variety of different statistical and machine learning algorithms that can be adopted to reach this goal, ranging from simple strategies that assume little or nothing about the nature of the phenomenon to more complex ones that involve multiple features. Our approach to time series forecasting has been to privilege machine learning algorithms because they do not require any prior knowledge on the nature of the data distribution [11] and allow for more flexibility to integrate additional variables as predictors. There are several studies that confirm how machine learning algorithms have become increasingly popular in dealing with time series forecasting tasks [12, 11, 13, 14, 15].

In this study, we chose to use the *eXtreme Gradient Boosting* (XGBoost) algorithm [16] (`https://xgboost.readthedocs.io/en/latest/`) that has been widely used in many fields to achieve state-of-the-art results on famous data challenges (e.g. Kaggle competitions). XGBoost belongs to a category of so called ensemble learning approaches, which is a branch of machine learning methods that train and predict with many models at once to produce a single better output. In the case of XGBoost, the base model is a decision tree that is considered best-in-class for handling small to medium-sized data. The XGBoost algorithm deals with supervised machine learning tasks, like classification and regression. In this regard, the time series forecast process must be adapted to the regression setup. Since the concerned configuration does not support a multi-output design, we employ 30 different regression models in order to cover all our prediction horizons.

6.1 Modeling framework

Supervised learning is a machine learning paradigm for acquiring relationship information between input features (X) and output targets (y). The goal of supervised learning is to build an artificial system that can learn the mapping between the input and the output. These points with which the algorithm is trained are called training points.

The core of forecasting is knowledge of the past. Therefore, the formulation of the related supervised learning problem causes the input features and the output targets to *look back* in history, inspecting how the past has affected the near future. The basic concept is that an appropriate subset of temporal lags, starting from a historical reference date, explains the close evolution of the phenomenon investigated in a particular system.

In this study, we deal with systems representing the administrative regions of different countries. Each of these regions is described by different time series concerning food insecurity, as described in the *Data Sources* section. We need to pay attention to the heterogeneity of our systems: the multiple indicators translate into multivariate time series with different temporal resolutions.

The construction of the input-output samples relies on rolling a temporal window over the time series in order to collect a set of inputs based on predefined temporal lags and a set of outputs based on prediction horizons.

Let's suppose to analyze a system described by three short time series (A, B and

C) of which we want to predict one of them (A). These time series have different temporal resolutions: 1 day, 3 days and 5 days respectively. As described above, we need to define some temporal lags for these time series (e.g. $A : [1, 2, 3, 5]; B : [1, 2]; C : [1, 2, 3]$). Following the rolling scheme, we can obtain a set of training points for the first prediction horizon ($h = 1$), as shown in Figs. 21a and 21b.

Supplementary Figure 21: **Supervised learning conversion.** These tables show some examples of input features (blue cells) and output targets (orange cells) for two different prediction horizons.

Time-series	A	B	C
2018-08-22	1.0	1.0	nan
2018-08-23	2.0	nan	nan
2018-08-24	3.0	nan	3.0
2018-08-25	4.0	4.0	nan
2018-08-26	5.0	nan	nan
2018-08-27	6.0	nan	nan
2018-08-28	7.0	7.0	nan
2018-08-29	8.0	nan	8.0
2018-08-30	10.0	nan	nan
2018-08-31	11.0	11.0	nan
2018-09-01	12.0	nan	nan
2018-09-02	13.0	nan	nan
2018-09-03	14.0	14.0	14.0
2018-09-04	15.0	nan	nan
2018-09-05	16.0	nan	nan
2018-09-06	17.0	17.0	nan
2018-09-07	18.0	nan	nan
2018-09-08	19.0	nan	19.0
2018-09-09	20.0	20.0	nan
2018-09-10	21.0	nan	nan
2018-09-11	22.0	nan	nan
2018-09-12	23.0	23.0	nan
2018-09-13	24.0	nan	24.0
2018-09-14	25.0	nan	nan
2018-09-15	26.0	26.0	nan
2018-09-16	27.0	nan	nan
2018-09-17	28.0	nan	nan
2018-09-18	29.0	29.0	29.0

(a) First training sample (X, y) for the prediction horizon $h = 1$.

Time-series	A	B	C
2018-08-22	1.0	1.0	nan
2018-08-23	2.0	nan	nan
2018-08-24	3.0	nan	3.0
2018-08-25	4.0	4.0	nan
2018-08-26	5.0	nan	nan
2018-08-27	6.0	nan	nan
2018-08-28	7.0	7.0	nan
2018-08-29	8.0	nan	8.0
2018-08-30	10.0	nan	nan
2018-08-31	11.0	11.0	nan
2018-09-01	12.0	nan	nan
2018-09-02	13.0	nan	nan
2018-09-03	14.0	14.0	14.0
2018-09-04	15.0	nan	nan
2018-09-05	16.0	nan	nan
2018-09-06	17.0	17.0	nan
2018-09-07	18.0	nan	nan
2018-09-08	19.0	nan	19.0
2018-09-09	20.0	20.0	nan
2018-09-10	21.0	nan	nan
2018-09-11	22.0	nan	nan
2018-09-12	23.0	23.0	nan
2018-09-13	24.0	nan	24.0
2018-09-14	25.0	nan	nan
2018-09-15	26.0	26.0	nan
2018-09-16	27.0	nan	nan
2018-09-17	28.0	nan	nan
2018-09-18	29.0	29.0	29.0

(b) Last training sample (X, y) for the prediction horizon $h = 1$.

Time-series	A	B	C
2018-08-22	1.0	1.0	nan
2018-08-23	2.0	nan	nan
2018-08-24	3.0	nan	3.0
2018-08-25	4.0	4.0	nan
2018-08-26	5.0	nan	nan
2018-08-27	6.0	nan	nan
2018-08-28	7.0	7.0	nan
2018-08-29	8.0	nan	8.0
2018-08-30	10.0	nan	nan
2018-08-31	11.0	11.0	nan
2018-09-01	12.0	nan	nan
2018-09-02	13.0	nan	nan
2018-09-03	14.0	14.0	14.0
2018-09-04	15.0	nan	nan
2018-09-05	16.0	nan	nan
2018-09-06	17.0	17.0	nan
2018-09-07	18.0	nan	nan
2018-09-08	19.0	nan	19.0
2018-09-09	20.0	20.0	nan
2018-09-10	21.0	nan	nan
2018-09-11	22.0	nan	nan
2018-09-12	23.0	23.0	nan
2018-09-13	24.0	nan	24.0
2018-09-14	25.0	nan	nan
2018-09-15	26.0	26.0	nan
2018-09-16	27.0	nan	nan
2018-09-17	28.0	nan	nan
2018-09-18	29.0	29.0	29.0

(c) First training sample (X, y) for the prediction horizon $h = 3$.

These training points are pairs of input features (X) and output targets (y) that allow the XGBoost model to predict one day into the future. The input features also contain temporal information regarding the current prediction horizon. Date-time based features are a good solution in providing a temporal dimension to the regression models. All the training points of this example are shown in Figures 22.

The creation of the training points related to the prediction horizon $h = 3$ follows the same criteria. The main difference regards the temporal gap between the latest date of each input sample and the corresponding output sample, as shown in Fig. 21c.

We apply this method to our task by analyzing each administrative region of our countries. More precisely, each region is characterized by a common set of indicators that translate into training points with the same structure. The heterogeneity of the systems is managed by preserving the temporal granularity of the time series among the various indicators during the rolling phase. At the end of the process, we obtain

Supplementary Figure 22: **Training points.** These tables show the input features (a) and output targets (b) for the prediction horizon $h = 1$.

Features	A				B		C			Day	Month	Year
Lags	x(t-4)	x(t-2)	x(t-1)	x(t)	x(t-1)	x(t)	x(t-2)	x(t-1)	x(t)	x(t+1)	x(t+1)	x(t+1)
0	10.0	12.0	13.0	14.0	11.0	14.0	3.0	8.0	14.0	4	9	2018
1	11.0	13.0	14.0	15.0	11.0	14.0	3.0	8.0	14.0	5	9	2018
2	12.0	14.0	15.0	16.0	11.0	14.0	3.0	8.0	14.0	6	9	2018
3	13.0	15.0	16.0	17.0	14.0	17.0	3.0	8.0	14.0	7	9	2018
4	14.0	16.0	17.0	18.0	14.0	17.0	3.0	8.0	14.0	8	9	2018
5	15.0	17.0	18.0	19.0	14.0	17.0	8.0	14.0	19.0	9	9	2018
6	16.0	18.0	19.0	20.0	17.0	20.0	8.0	14.0	19.0	10	9	2018
7	17.0	19.0	20.0	21.0	17.0	20.0	8.0	14.0	19.0	11	9	2018
8	18.0	20.0	21.0	22.0	17.0	20.0	8.0	14.0	19.0	12	9	2018
9	19.0	21.0	22.0	23.0	20.0	23.0	8.0	14.0	19.0	13	9	2018
10	20.0	22.0	23.0	24.0	20.0	23.0	14.0	19.0	24.0	14	9	2018
11	21.0	23.0	24.0	25.0	20.0	23.0	14.0	19.0	24.0	15	9	2018
12	22.0	24.0	25.0	26.0	23.0	26.0	14.0	19.0	24.0	16	9	2018
13	23.0	25.0	26.0	27.0	23.0	26.0	14.0	19.0	24.0	17	9	2018
14	24.0	26.0	27.0	28.0	23.0	26.0	14.0	19.0	24.0	18	9	2018

(a) Input features X.

Target	A
Prediction horizon	x(t+1)
0	15.0
1	16.0
2	17.0
3	18.0
4	19.0
5	20.0
6	21.0
7	22.0
8	23.0
9	24.0
10	25.0
11	26.0
12	27.0
13	28.0
14	29.0

(b) Output targets y.

a set of training points for each prediction horizon and region. The choice of the lagged values for our indicators is reported in Table 7 and is led by the knowledge that long history lengths do not benefit the prediction.

Supplementary Table 7: The lags values for each indicator.

	Insufficient food consumption	Crisis or above food-based coping	Fatalities	1 month rainfall anomaly	NDVI	NDVI anomaly	Rainfall	Market Prices	Ramadan
**SYR**	1-13	1	5	1	/	1	1	1	1
**BFA**	1-12	1-7	1	1	/	1	1	1	1
**CMR**	1-12	1	1	1	/	1	1	1	1
**MLI**	1-13	1	1	1	/	1	1	1	1
**YEM**	1-10	5	1	1	/	1	1	1	1
**NGA**	1-12	6	1	1	1	1	1	1	1

6.2 Model Evaluation

The scheme of our approach is the following:

1. Suppose we go back in time and find ourselves at the end of January 2020 with knowledge of the data history limited to that date. Our current aim is to predict food insecurity for the following 30 days;
2. On the basis of what was known until the end of January 2020, XGBoost can be trained to predict food insecurity in the following 30 days;
3. Suppose that time passes and we reach the end of February 2020. With the updating of new real data, we can test the generated prediction;

4. Now, we would like to predict food insecurity in the following 30 days from the end of February 2020;
5. And so on until we reach the end of August 2020.

In this way, we get 7 unbiased *splits* that simulate an application of our solution over time for each administrative region, as shown in Fig. 23.

Supplementary Figure 23: **Nested cross-validation.** The illustrative scheme of nested cross-validation used in this study.

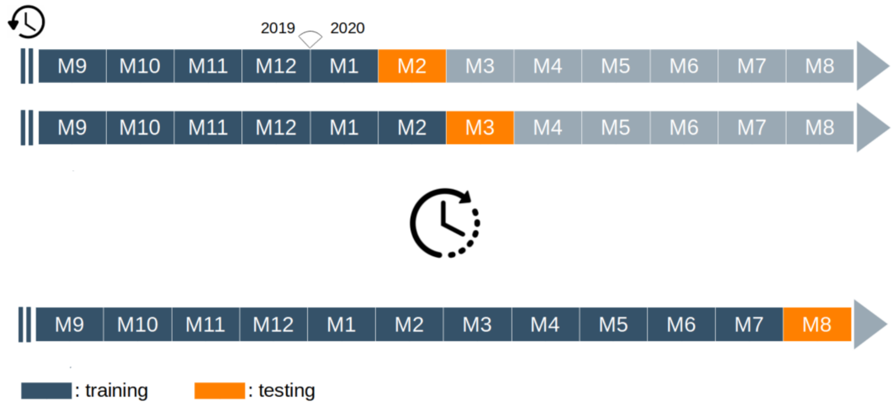

The validation phase fits into this process by simply splitting the training points of each region into two sets: the first 80% samples are used for training and the remaining ones for validation. Validation is performed independently between the splits ensuring the use of an unbiased approach, as shown in Fig. 24.

Our validation scheme aims to optimize the prediction framework by both tuning model hyper-parameters and performing feature selection. The aim of this optimization is to find the configuration that returns the best performance as measured on a validation set. It can be performed following four common methods: manual, grid search, random search and bayesian model-based optimization. All these techniques are based on the minimization of an objective function. In simple terms, we want to find the configuration that yields the best objective score on the validation set. The problem with the optimization is that evaluating the objective function to find the score is extremely expensive. Each time we try different configurations, we have to train a model on the training data, make predictions on the validation data, and then calculate the objective score. Grid search and random search are the most popular optimization techniques. However, these methods are relatively inefficient because they do not explore the space of the configurations evaluating previous iterative results. Grid and random search are completely uninformed by past evaluations, and as a result, often spend a significant amount of time evaluating bad configurations. For this reason, we focus our attention on the bayesian approach that keeps track of past evaluation results. In this regard, we integrate into our model the Hyperopt library [17] which intelligently explores the search space while narrowing down to the estimated best configurations. The objective function that we decided to use is a trade-off estimation between the R^2 metrics on the training and validation sets:

$$L = |R^2_{train} - R^2_{validation}| \cdot w_1 + (1 - R^2_{train}) \cdot w_2 \tag{3}$$

Supplementary Figure 24: **Model Evaluation.** (a) The training, validation and test sets for a generic split. (b) The overall scheme of evaluation among the splits.

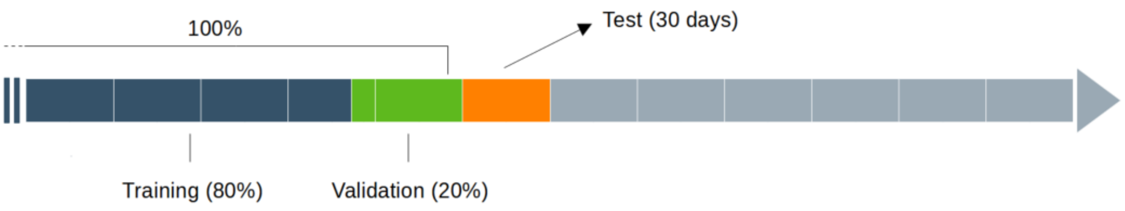

(a) Training, validation and test.

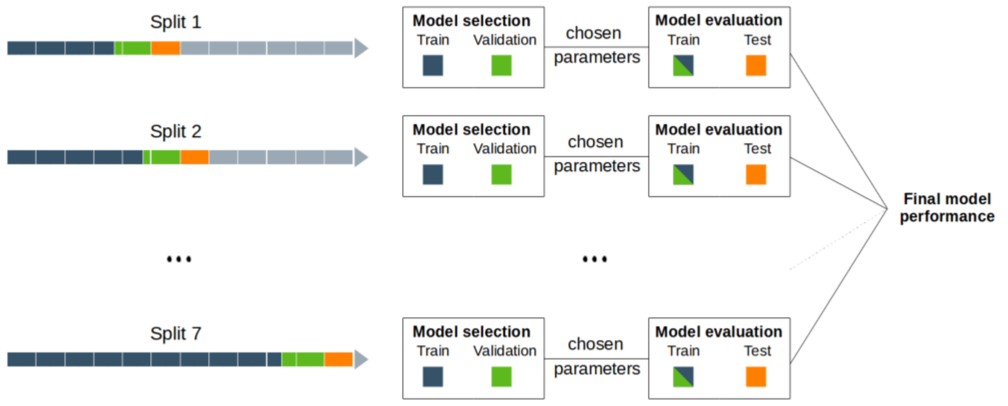

(b) Evaluation scheme.

where $w_1 = 0.7$ and $w_2 = 0.3$. The first part of the equation is meant to select the model that minimizes the difference in R^2 between training and validation, in order to minimize overfitting. The second part allows to ensure that only models with high enough R^2 are selected.

Moreover, an early stopping occurs when no more advantage are measured on the validation set in order to prevent overfitting and computational costs.

More in details, our hyper-parameter optimization explores the set of XGBoost parameters and corresponding values reported in Table 8.

Supplementary Table 8: The hyper-parameters of the XGBoost model that are optimized.

	**Start**	**End**	**Step**
**gamma**	0.0	1.0	0.005
**n_estimators**	1000	/	/
**reg_alpha**	0.0	1.0	0.005
**reg_lambda**	0.0	1.0	0.005
**max_depth**	3	6	1
**min_child_weight**	1	10	0.1
**learning_rate**	0.001	0.3	0.001
**subsample**	0.6	1.0	0.05
**colsample_bytree**	0.6	1.0	0.05

In regards to the indicators selection, we explore different configurations of the

independent variables deciding to keep or exclude some based on the results on the validation set. For each split, the starting configuration uses the default parameters of the XGBoost model and the univariate setting where the only predictor is the target variable. The total number of hyperopt configurations is 600 for each country.

References

[1] WFP. *Food consumption analysis Calculation and use of the food consumption score in food security analysis*. 2008. URL: https://documents.wfp.org/stellent/groups/public/documents/manual_guide_proced/wfp197216.pdf.

[2] WFP. *The Coping Strategies Index: Field Methods Manual*. 2008. URL: https://documents.wfp.org/stellent/groups/public/documents/manual_guide_proced/wfp211058.pdf.

[3] IPC Global Partners. *Integrated Food Security Phase Classification: Technical Manual Version 3.0: Evidence and Standards for Better Food Security Decisions*. Food and Agriculture Organization of the United Nations, 2019.

[4] Clionadh Raleigh, Andrew Linke, Håvard Hegre, and Joakim Karlsen. "Introducing ACLED: An Armed Conflict Location and Event Dataset: Special Data Feature". In: *Journal of Peace Research* 47 (Sept. 2010), pp. 651–660.

[5] Chris C Funk, Pete J Peterson, Martin F Landsfeld, Diego H Pedreros, James P Verdin, James D Rowland, Bo E Romero, Gregory J Husak, Joel C Michaelsen, Andrew P Verdin, et al. "A quasi-global precipitation time series for drought monitoring". In: *US Geological Survey data series* 832.4 (2014), pp. 1–12.

[6] *MODIS Vegetation Index Products (NDVI and EVI)*. https://modis.gsfc.nasa.gov/data/dataprod/mod13.php.

[7] Samuel Scarpino and Giovanni Petri. "On the predictability of infectious disease outbreaks". In: *Nature Communications* 10 (Feb. 2019).

[8] Javier Borge-Holthoefer, Nicola Perra, Bruno Gonçalves, Sandra González-Bailón, Alex Arenas, Yamir Moreno, and Alessandro Vespignani. "The Dynamics of Information-Driven Coordination Phenomena: A Transfer Entropy Analysis". In: *Science Advances* 2 (July 2015).

[9] Liangyue Cao. "Practical method for determining the minimum embedding dimension of a scalar time series". In: *Physica D: Nonlinear Phenomena* 110.1 (1997), pp. 43–50. ISSN: 0167-2789.

[10] Matthew B. Kennel, Reggie Brown, and Henry D. I. Abarbanel. "Determining embedding dimension for phase-space reconstruction using a geometrical construction". In: *Phys. Rev. A* 45 (6 Mar. 1992), pp. 3403–3411.

[11] Antonio Rafael Sabino Parmezan, Vinicius M.A. Souza, and Gustavo E.A.P.A. Batista. "Evaluation of statistical and machine learning models for time series prediction: Identifying the state-of-the-art and the best conditions for the use of each model". In: *Information Sciences* 484 (2019), pp. 302–337. ISSN: 0020-0255. DOI: https://doi.org/10.1016/j.ins.2019.01.076. URL: https://www.sciencedirect.com/science/article/pii/S0020025519300945.

[12] Antonio Rafael Sabino Parmezan and Gustavo E.A.P.A. Batista. "A Study of the Use of Complexity Measures in the Similarity Search Process Adopted by kNN Algorithm for Time Series Prediction". In: *2015 IEEE 14th International Conference on Machine Learning and Applications (ICMLA)*. 2015, pp. 45–51. DOI: 10.1109/ICMLA.2015.217.

[13] Renzhuo Wan, Shuping Mei, Jun Wang, Min Liu, and Fan Yang. "Multivariate Temporal Convolutional Network: A Deep Neural Networks Approach for Multivariate Time Series Forecasting". In: *Electronics* 8 (Aug. 2019), p. 876.

[14] Alaa Sagheer and Mostafa Kotb. "Unsupervised Pre-training of a Deep LSTM-based Stacked Autoencoder for Multivariate Time Series Forecasting Problems". In: *Scientific Reports* 9 (Dec. 2019), p. 19038.

[15] Haibin Cheng, Pang-Ning Tan, Jing Gao, and Jerry Scripps. "Multistep-Ahead Time Series Prediction". In: *Advances in Knowledge Discovery and Data Mining*. Ed. by Wee-Keong Ng, Masaru Kitsuregawa, Jianzhong Li, and Kuiyu Chang. Berlin, Heidelberg: Springer Berlin Heidelberg, 2006, pp. 765–774.

[16] Tianqi Chen and Carlos Guestrin. "XGBoost: A Scalable Tree Boosting System". In: *Proceedings of the 22nd ACM SIGKDD International Conference on Knowledge Discovery and Data Mining*. KDD '16. San Francisco, California, USA: ACM, 2016, pp. 785–794. ISBN: 978-1-4503-4232-2. DOI: 10.1145/2939672.2939785. URL: http://doi.acm.org/10.1145/2939672.2939785.

[17] James Bergstra, Brent Komer, Chris Eliasmith, Dan Yamins, and David Cox. "Hyperopt: A Python library for model selection and hyperparameter optimization". In: *Computational Science & Discovery* 8 (July 2015), p. 014008.
